# A comparison of the Muenster, SIOP Boston, Brock, Chang and CTCAEv4.03 ototoxicity grading scales applied to 3,799 audiograms of childhood cancer patients treated with platinum-based chemotherapy

**DOI:** 10.1371/journal.pone.0210646

**Published:** 2019-02-14

**Authors:** E. Clemens, B. Brooks, A. C. H. de Vries, M. van Grotel, M. M. van den Heuvel-Eibrink, B. Carleton

**Affiliations:** 1 Princess Máxima Center for Pediatric Oncology, Utrecht, the Netherlands; 2 Department of Pediatric Hematology and Oncology, Erasmus MC – Sophia Children’s Hospital, Rotterdam, the Netherlands; 3 Audiology and Speech Pathology Department, British Columbia Children’s Hospital, Vancouver, British Columbia, Canada; 4 BC Children’s Hospital Research Institute, Vancouver, British Columbia, Canada; 5 Division of Translational Therapeutics, Department of Pediatrics, The University of British Columbia, Vancouver, British Columbia, Canada; 6 Pharmaceutical Outcomes Programme, BC Children’s Hospital, Vancouver, British Columbia, Canada; University of Bern, SWITZERLAND

## Abstract

Childhood cancer patients treated with platinums often develop hearing loss and the degree is classified according to different scales globally. Our objective was to compare concordance between five well-known ototoxicity scales used for childhood cancer patients. Audiometric test results (n = 654) were evaluated longitudinally and graded according Brock, Chang, International Society of Pediatric Oncology (SIOP) Boston, Muenster scales and the U.S. National Cancer Institute Common Technology Criteria for Adverse Events (CTCAE) version 4.03. Adverse effects of grade 2, 3 and 4 are considered to reflect a degree of hearing loss sufficient to interfere with day-to-day communication (> = Chang grade 2a; > = Muenster grade 2b). We term this “deleterious hearing loss”. A total number of 3,799 audiograms were evaluated. The prevalence of deleterious hearing loss according to the last available audiogram of each patient was 59.3% (388/654) according to Muenster, 48.2% (315/653) according to SIOP, 40.5% (265/652) according to Brock, 40.3% (263/652) according to Chang, and 57.5% (300/522) according to CTCAEv4.03. Overall concordance between the scales ranged from ĸ = 0.636 (Muenster vs. Chang) to ĸ = 0.975 (Brock vs. Chang). Muenster detected hearing loss the earliest in time, followed by Chang, SIOP and Brock. Generally good concordance between the scales was observed but there is still diversity in definitions of functional outcomes, such as differences in distribution levels of severity of hearing loss, and additional intermediate scales taking into account losses <40 dB as well. Regardless of the scale used, hearing function decreases over time and therefore, close monitoring of hearing function at baseline and with each cycle of platinum therapy should be conducted.

## Introduction

Cisplatin and carboplatin are platinum compounds commonly used in the treatment of pediatric malignancies. Ototoxicity, a common adverse effect of treatment with cisplatin, is initially manifested as high frequency hearing loss commonly accompanied by tinnitus[1–4]. Hearing impairment in children can enhance learning problems by influencing speech and language development as children have difficulty understanding speech and sounds, especially in the presence of ambient noise[5, 6]. The overall reported incidence of platinum-induced ototoxicity in children approaches 60%[1, 7, 8] and is influenced by younger age, higher cumulative cisplatin or cranial radiotherapy doses, and concomitant treatment with aminoglycosides and furosemide[9–12]. Although carboplatin is considered less ototoxic than cisplatin, hearing impairment can occur, especially with high doses[13–15]. Besides treatment and patient-related factors, perception of the seriousness of ototoxicity is also influenced by the variability in the criteria that are used to indicate degree of ototoxicity[16, 17]. Although all criteria used to assign hearing loss grades do so on scales ranging from no hearing loss to severe hearing loss, there is significant variability in the design and criteria included within the grading systems.

Hearing status is usually measured by audiometry up to 8 kHz. For surveillance of hearing loss, the 5-point U.S. National Cancer Institute Common Terminology Criteria for Adverse Events (CTCAE) scale is the most commonly used classification to grade hearing loss (Table 1 and S1A Fig)[18]. Most clinicians are familiar with the interpretation of this grading system. CTCAE grading is designed to identify adverse events that require clinical action, and is aligned with the Medical Dictionary for Regulatory Activities (medDRA) to assign grades based on expected severity of impact on physical functioning and daily living activities.

**Table 1:** Ototoxicity classification systems.

CTCAEv4.03	Muenster	SIOP	Brock	Chang	Deleterious hearing loss
**Grade 0**: <20 dB all	**Grade 0**: ≤10 dB all	**Grade 0**: ≤20 dB all	**Grade 0**: <40 dB all	**Grade 0**: ≤20 dB at 1, 2 and 4 kHz	**No**
**Grade 1**: >20 dB at 8 kHz	**Grade 1**: >10 dB at ≤2 kHz	**Grade 1**: >20 dB at >4 kHz	**Grade 1**: ≥40 dB at 8 kHz	**Grade 1a**: ≥40 dB at 6–12 kHz	
	**Grade 2a**: >20 ≤40 dB at ≥4 kHz			**Grade 1b**: >20 dB and <40 dB at 4 kHz	
**Grade 2**: >20 dB at ≥4 kHz *(if 6 kHz measured*, *use 6 kHz)*	**Grade 2b**: >40 ≤60 dB at ≥4 kHz	**Grade 2**: >20 dB at ≥4 kHz	**Grade 2**: ≥40 dB at ≥4 kHz	**Grade 2a**: ≥40 dB at ≥4 kHz	**Yes**
**Grade 3**: >20 dB at ≥3 kHz *(if 3 kHz not measured*, *use 2 kHz)*	**Grade 2c**: >60 dB at ≥4 kHz	**Grade 3**: >20 dB at 2 or 3 kHz	**Grade 3**: ≥40 dB at ≥2 kHz	**Grade 2b**: >20 and <40 dB at 1, 2 or 3 kHz	
**Grade 4**: Audiological indication for cochlear implant: ≥50 dB at ≥1 kHz	**Grade 3a**: >20 ≤40 dB at <4 kHz	**Grade 4**: >40 dB at ≥2 kHz	**Grade 4**: ≥40 dB at ≥1 kHz	**Grade 3**: ≥40 dB at ≥2 or 3 kHz	
	**Grade 3b**: >40 ≤60 dB at <4 kHz			**Grade 4**: ≥40 dB at ≥1 kHz	
	**Grade 3c**: >60 <80 dB at <4 kHz				
	**Grade 4**: ≥80 dB at <4 kHz				

The Brock grading system is a 5-point scale and was specifically designed for pediatric patients treated with cisplatin. This system is used to grade hearing loss progression from high to lower frequencies, taking into account the typical cisplatin-induced configuration of high frequency loss (Table 1 and S1B Fig)[19]. Hearing loss is classified according to the frequency range where hearing thresholds exceed 40 dB. It is important to acknowledge that hearing losses up to 40 dB at any frequency are not considered by Brock criteria and therefore, a Brock grade 0 does not imply normal hearing status.

A modification of the Brock scale resulted in the more clinically-sensitive Chang criteria that correlated with the expected course of treatment-induced ototoxicity in clinical trials (Table 1 and S1C Fig)[20]. This scale classifies the loss based on a 7-point scale and accounts for any hearing loss less than 40 dB at any frequency, thereby resulting in a greater clinical sensitivity. This scale also includes sub-scales (grade 1a, 1b, 2a and 2b) to capture hearing thresholds in the mild loss range of 20 to 40 dB at all frequencies.

The 8-point Muenster scale is based on the World Health Organization classification and includes several subgroups (grade 2a, 2b, 2c, 3a, 3b and 3c)[21]. This grading system was designed for early detection of hearing loss (Table 1 and S1D Fig).

More recently, the International Society of Pediatric Oncology (SIOP) Boston classification was released through international consensus to report hearing outcomes in international clinical trials for pediatric patients treated with platinum therapy (Table 1 and S1E Fig)[16]. This is a 5-point scale, adopting components of the Brock, CTCAE, Chang and Muenster grading scales and taking into account the functional outcome of a patient at the end of treatment.

Audiograms can be graded for degree of hearing loss according to the hearing status, based on the audiogram alone. Alternatively, audiological datasets including additional information such as immittance, otoacoustic emission, and speech audiometry findings as well as patient history can be examined to determine the likelihood and extent of ototoxicity. The latter is important when an adverse drug effect is suspected and contribution to debilitation of additional etiologies must be eliminated. The objectives of this study are to summarize the baseline characteristics of a large cohort of pediatric childhood cancer patients treated with platinum compounds, and to compare five grading systems within this cohort.

### Patients and methods

The analysis of the anonymized Canadian cohort was approved by the ethics committees of the University of British Columbia and British Columbia’s Children’s Hospital. The analysis of the anonymized Dutch cohort was approved by the ethics committee of the Erasmus Medical Center (MEC-2015-169).

### Study population

Patients were recruited from the BC Children’s Hospital (Vancouver, Canada), Alberta Children’s Hospital (Calgary, Canada), Stollery Children’s Hospital (Edmonton, Vancouver), Winnipeg Health Sciences Centre (Winnipeg, Canada), London Health Sciences Center (London, Canada), Hospital for Sick Children (Toronto, Canada), Kingston General Hospital (Kingston, Canada), Children’s Hospital of Eastern Ontario (Ottawa, Canada), Hôpital Saint-Justine (Montreal, Canada), IWK Health Center (Halifax, Canada), McMaster Children’s Hospital (Hamilton, Canada), and the Erasmus Medical Center—Sophia Children’s Hospital (Rotterdam, the Netherlands). Ethical approval was obtained from local research ethics boards. Serial audiological data were collected from 729 patients who received platinum chemotherapy between November 1981 and November 2016. Patients with previous hearing problems (n = 1) or missing data were excluded (n = 74). A total number of 654 patients were included and the total number of audiological evaluations was 3,799. Characteristics of the 654 patients studied are listed in Table 2. Age at start treatment ranged between 0 and 18.8 years (median: 5.4 years) and age at recent evaluation ranged between 1.2 and 35.6 years (median: 12.6 years). The majority of children were treated for germ cell tumor, neuroblastoma, osteosarcoma and brain tumor. Forty-four patients were treated with carboplatin, 469 patients were treated with cisplatin alone, and 141 were treated with cisplatin and additional carboplatin. Ninety-eight (15%) patients received additional cranial radiotherapy.

**Table 2:** Baseline characteristics of included patients.

	All patients (N = 654)
**Sex, n (%)**	
Male	318 (48.6)
Female	335 (51.2)
Missing	1 (0.2)
**Age at start treatment, median years (n (%))**	5.4 (0.05–18.8)
**Age at recent evaluation, median years (n (%))**	12.6 (1.2–35.6)
**Carboplatin, n (%)**	185 (28.3)
**Cumulative cisplatin dose, mg/m**^**2**^ **(range)**	400 (55–1600)
**Cumulative carboplatin dose, mg/mg**^**2**^ **(range)**	1700 (250–9436)
**Cranial radiotherapy, n (%)**	98 (18.8)
**Diagnosis, n (%)**	
Brain tumor	113 (17.3)
Carcinoma	11 (1.7)
Germ cell tumor	140 (21.4)
Lymphoma	6 (0.9)
Neuroblastoma	128 (19.6)
Osteosarcoma	119 (18.2)
Other malignancies	9 (1.4)
Retinoblastoma	1 (0.2)
Rhabdomyosarcoma	21 (3.2)
Soft tissue sarcoma	2 (0.4)

### Outcome measures and definitions of ototoxicity

Serial audiogram data was available from pure tone audiometry at frequencies of 0.5, 1, 2, 3, 4, 6, and 8 kHz. Testing was performed by the pediatric audiologists. For the first method, we retrospectively graded 3,789 audiograms from Canadian and Rotterdam patients for overall degree of sensorineural hearing loss according to the following grading systems:, the Muenster[21], the SIOP[16], Brock[19] and Chang[20] grading scales (Table 1). In case of asymmetric hearing loss, the worst hearing ear was used for analysis. For the second method, CTCAE grading was used to phenotype for ototoxicity for 522 Canadian patients based on their serial audiological data sets including any available additional data such as immittance tests, otoacoustic emissions, extended high frequency data and audiologist’s narrative in addition to the audiograms[18]. The CTCAE criteria specify baseline evaluations to calculate the threshold shifts. Because absolute hearing threshold baseline data was not available for many of the children, absolute threshold levels were used instead of changes from baseline. When 6 kHz data was available, it was used to differentiate grade 1 from grade 2; that is, a patient would be assigned “grade 2” if the hearing threshold was greater than 20 dB at 6 kHz and at all frequencies tested above 6 kHz and the remainder of the audiologic dataset supported an ototoxic effect as the most likely etiology. Further, in the definition of grade 4, “indication for cochlear implant and speech-language services” was specified as “hearing threshold of greater than or equal to 50 dB at 1 kHz and frequencies above”. If middle ear pathology or conductive hearing loss was present, audiological grading was based on bone conduction thresholds. If bone conduction thresholds were not obtained and there was indication of middle ear pathology, the assessment was categorized as not evaluable. Demographics, cancer diagnosis and treatment exposures were extracted from the medical records.

For the CTCAEv4.03, Brock and SIOP Boston, children with deleterious hearing loss were defined as those with grade 2 or greater. For Muenster, patients were classified as having deleterious hearing loss if the grade was 2b or greater. For Chang, children with deleterious hearing loss were defined as those with grade 2a or greater. To compare the longitudinal data of audiometric testing, we displayed the results of different ototoxicity classification systems. Due to the absence of standardized measuring timepoints after the start of treatment, adjacent measurements were combined at predetermined points. The prevalence of deleterious hearing loss was determined at the date of treatment discontinuation, one year after treatment discontinuation and 10 years after treatment discontinuation. Descriptive statistics included median and range for continuous variables, and frequencies including percentages for categorical variables.

### Statistical analysis

Two approaches were used to compare the different ototoxicity classification systems as the CTCAEv4.03 criteria were applied only on the last available audiogram of each patient. For the first analysis all available audiograms were compared and for the second analysis the ototoxicity scales of the last available audiogram of each patient were compared. Κ statistics were estimated and we consider a clinically meaningful ĸ to be at least 0.70. All analyses were performed using SPSS 24.0 software (SPSS Inc., Chicago, IL, USA).

## Results

### Prevalence of hearing loss

Evaluation of the first post treatment audiogram showed that 282/549 patients (51.4%) had deleterious hearing loss according to Muenster, 231/548 (42.2%) according to SIOP, 182/549 (33.2%) according to Brock and 188/548 (34.3%) according to Chang. Median time after the end of platinum treatment was 0.3 years (range: 1 day–26.2 years). The prevalence of ototoxicity one year after treatment discontinuation (median: 1.38 years; range: 1.1–2.0 years) was 43% according to Brock and Chang, 56% according to Muenster and 49% according to SIOP. Ten years after treatment discontinuation (range: 9.01–10.98), the prevalence of ototoxicity was 46% according to Brock and Chang, 61% according to Muenster and 54% according to SIOP (Table 3).

**Table 3:** Grade distribution (%) based on the last available audiogram according to different CTCAEv4.03, Muenster, SIOP, Brock and Chang classification systems.

Scale	Grade 0, n (%)	Grade 1a, n (%)	Grade 1b, n (%)	Grade 2a, n (%)	Grade 2b, n (%)	Grade 2c, n (%)	Grade 3a, n (%)	Grade 3b, n (%)	Grade 3c, n (%)	Grade 4, n (%)
**CTCAEv4.03**	171 (32.8)	50 (9.6)		133 (25.5)			153 (29.3)			14 (2.7)
**Muenster**	129 (19.7)	186 (13.3)		52 (7.8)	89 (13.6)	97 (14.8)	55 (8.4)	69 (10.7)	33 (5)	45 (6.9)
**SIOP**	215 (32.9)	123 (18.8)		103 (15.8)			109 (16.7)			103 (15.8)
**Brock**	255 (39.1)	132 (20.2)		153 (23.5)			77 (11.8)			35 (5.4)
**Chang**	249 (38.2)	96 (14.7)	44 (6.7)	59 (9)	38 (5.8)		130 (19.9)			36 (5.5)

Abbreviations: CTCAE = U.S. National Cancer Institute Common Technology Criteria for Adverse Events, SIOP = International Society of Pediatric Oncology Boston

**Fig 1:**
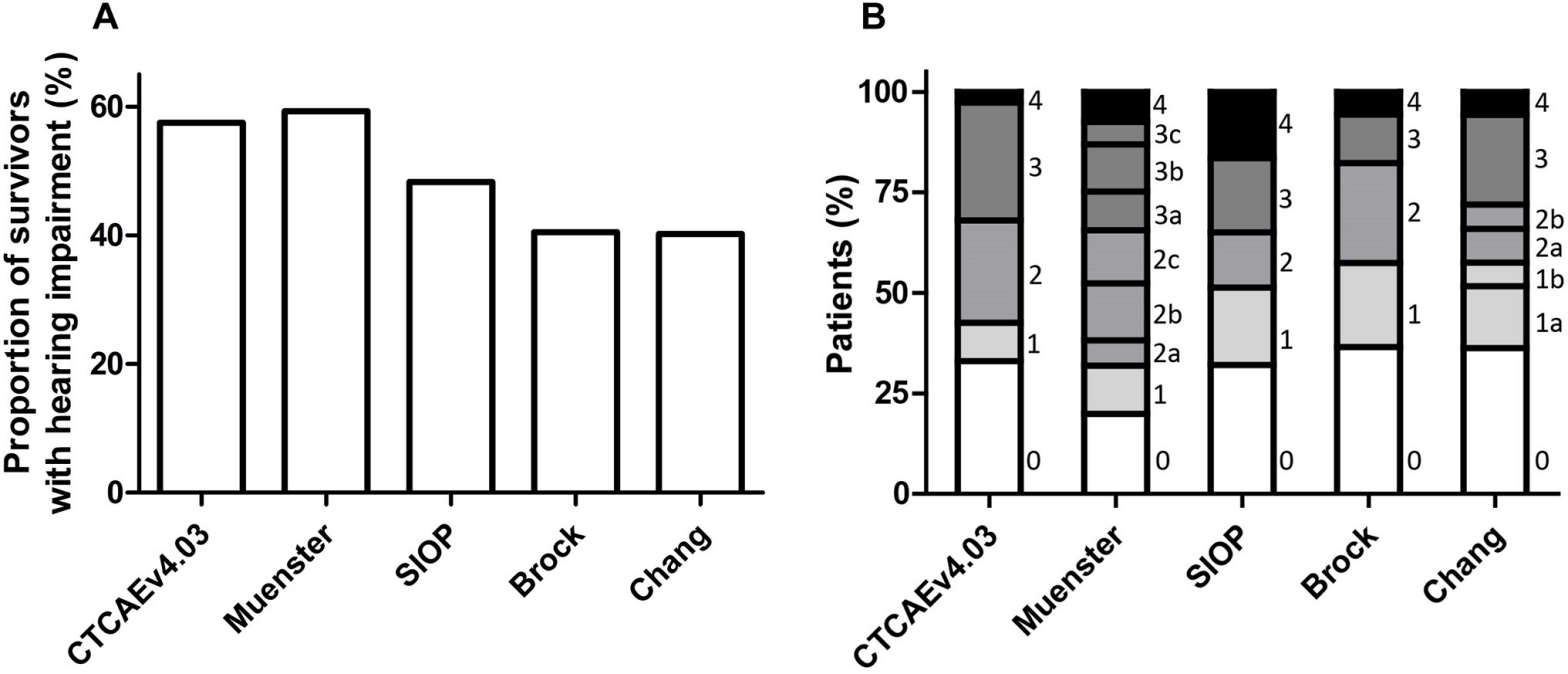
Prevalence of deleterious hearing loss (A) based on CTCAEv4.03 ≥grade 2, Muenster ≥grade 2b, SIOP ≥grade 2, Brock ≥grade 2 and Chang ≥grade 2a, and ototoxicity grade distribution (B) according to the last available audiogram.

There is debate about how much of a child’s high frequency ability can be damaged before the hearing loss has serious complications. For our comparison purposes we consider deleterious hearing loss for children, to be at grade 2 and higher degree on CTCAE, Brock and SIOP, grade 2b and higher on Muenster scale, and grade 2a and higher on Chang scale. As shown in Fig 1 and Table 4, the prevalence of deleterious hearing loss according to the last available audiogram was 57.5% (300/522) on CTCAEv4.03, 59.3% (388/654) on Muenster, 48.3% (316/653) on SIOP Boston, 40.5% (265/652) on Brock, and 40.2% (263/652) on Chang. The median time from end of cisplatin treatment to the last available evaluation was 4.5 years (range: -3–29.2 years).

**Table 4:** The prevalence of deleterious hearing loss at stop treatment, 1 year after stop and 10 years after stop treatment.

	Stop treatment	1 year after stop treatment	10 years after stop treatment
**Median years (range)**	0.25 (1 day–1 year)	1.38 (1.1–2.0)	10.04 (9.01–10.98)
**Muenster**	184/403 (46%)	150/268 (56%)	56/92 (61%)
**SIOP**	178/402 (44%)	130/268 (49%)	50/92 (54%)
**Brock**	152/402 (38%)	115/268 (43%)	42/92 (46%)
**Chang**	160/403 (40%)	116/268 (43%)	42/92 (46%)

### Concordance among audiological scales

The concordance among Muenster and Chang criteria was lowest (ĸ: 0.665) and the concordance among Chang and Brock criteria was highest (ĸ: 0.969, Table 5). Among the patients with deleterious hearing loss according to Brock, 98.3% also met this definition according to Muenster criteria and among the patients with deleterious hearing loss according to Muenster, 69.7% also met this definition according to Brock grading. Muenster grading considers patients to have deleterious hearing loss earlier than Brock. Among the patients with deleterious hearing loss according to Chang, 85.1% also have deleterious hearing loss according to Brock criteria and among the patients with deleterious hearing loss according to Brock, 99.2% also have deleterious hearing loss according to Chang.

**Table 5:** Concordance among different ototoxicity criteria.

***All audiograms***	
**Grading**	**Kappa (ĸ)**
Muenster vs. Chang	0.665
Muenster vs. Brock	0.666
Muenster vs. SIOP	0.739
SIOP vs. Brock	0.840
SIOP vs. Chang	0.850
Brock vs. Chang	0.969
***Last available audiogram***	
**Grading**	**Kappa (ĸ)**
Muenster vs. Chang	0.636
Muenster vs. Brock	0.641
CTCAEv4.03 vs. Brock	0.681
CTCAEv4.03 vs. Chang	0.681
Muenster vs. SIOP	0.744
CTCAEv4.03 vs. SIOP	0.797
SIOP vs. Chang	0.845
SIOP vs. Brock	0.851
CTCAEv4.03 vs. Muenster	0.857
Brock vs. Chang	0.975

Abbreviations: CTCAE = U.S. National Cancer Institute Common Technology Criteria for Adverse Events, SIOP = International Society of Pediatric Oncology Boston

When we compared the ototoxicity criteria of the most recent available audiograms, concordance among Muenster and Chang was lowest (ĸ: 0.636) and concordance among Chang and Brock was highest (ĸ: 0.975, Table 5). Among the patients with deleterious hearing loss according to Brock, 99.6% also meet this definition according to Muenster criteria and among the patients with deleterious hearing loss according to Muenster, 68.4% also met this criteria according to Brock grading. Among the patients with deleterious hearing loss according to Chang, 85.5% also had deleterious hearing loss according to Brock criteria. Among the patients with deleterious hearing loss according to Brock, 98.5% also had deleterious hearing loss according to Chang.

For grade 2, Brock, Chang and Muenster are similar in coding (hearing loss above or equal to 40 dB at 4 kHz and above, Table 1). Thirty-two percent of the audiograms assigned a Brock grade 2 were graded as Chang grade 2a (277/868, ĸ = 0.417) and 40% of the audiograms (345/868, ĸ = 0.180) were graded as Muenster grade 2b and 2c (S2 Fig). Of the 281 audiograms assigned a Chang grade 2a hearing loss, 277 (99%) were graded by Brock as grade 2 as well and 273 (97%) were graded as Muenster grade 2b and 2c (ĸ = 0.368). Thirty-six percent of the audiograms assigned a Muenster grade 2b and 2c were graded as Brock grade 2 (345/959) and 29% of the audiograms (273/959) were assigned a Chang grade 2a.

Chang grade 2b and SIOP grade 3 are similar in coding as well. Of the 231 audiograms assigned a Chang grade 2b, 211 (91%, ĸ = 0.407) were graded by SIOP grade 3, and of the 684 audiograms graded as SIOP grade 3, 211 (31%) were graded by Chang as grade 2b.

For grade 4 hearing loss, Brock and Chang are similar in coding as well. Of the 183 audiograms assigned a Brock grade 4 hearing loss, 181 (98.9%, ĸ = 0.945) were graded by Chang grade 4 as well, and of the 199 audiograms graded as Chang grade 4, 181 (91%) were graded by Brock as grade 4 as well.

### Detection of hearing loss over time

**Fig 2:**
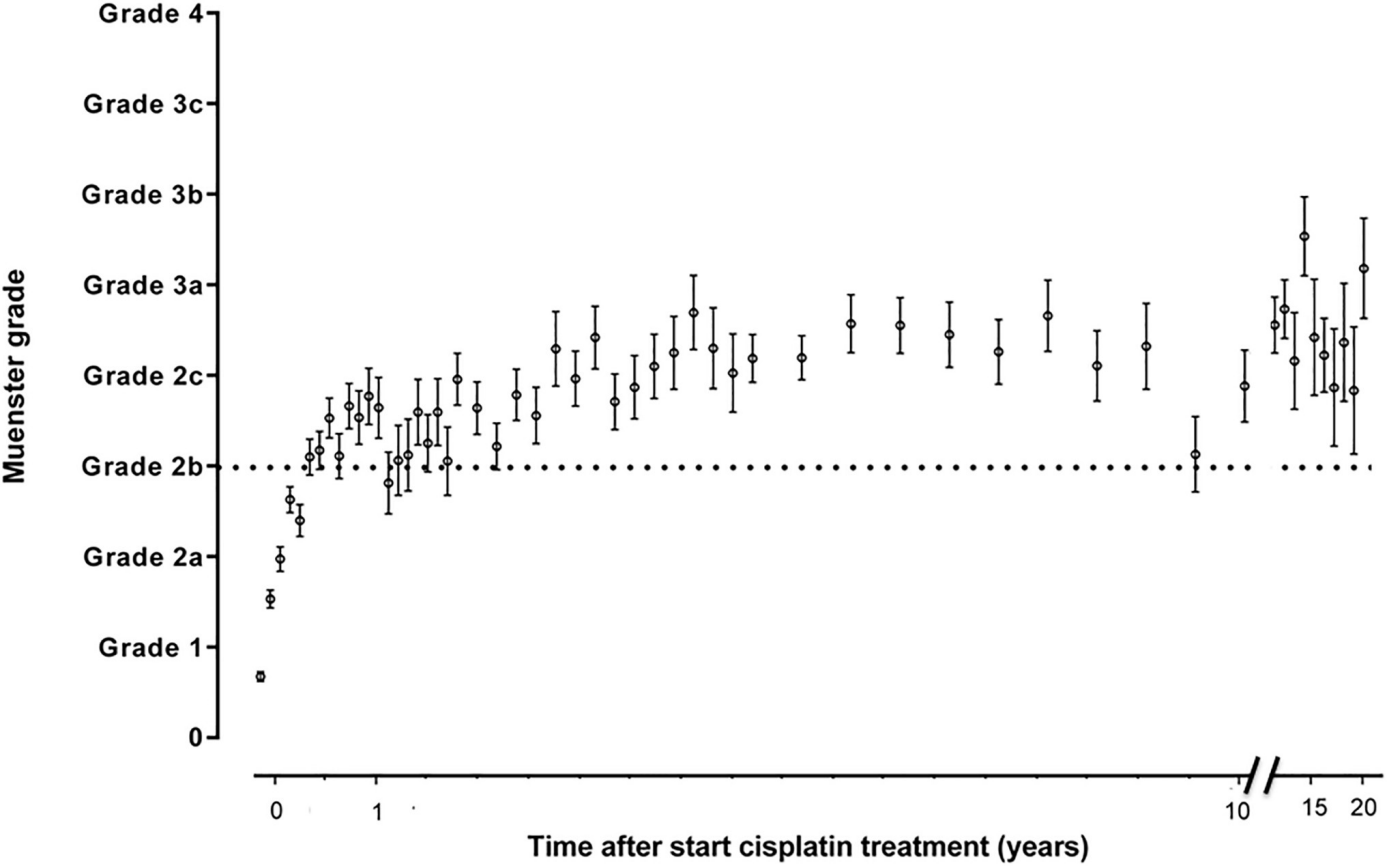
Progression of hearing loss based on Muenster grade. The time after start cisplatin treatment in 654 patients is depicted on the x-axis and the hearing loss grade is depicted on the y-axis. The dots represent the mean and the whiskers represent the standard error of the mean ototoxicity grade. The dotted horizontal line depicts the cut-off for hearing loss yes/no.

**Fig 3:**
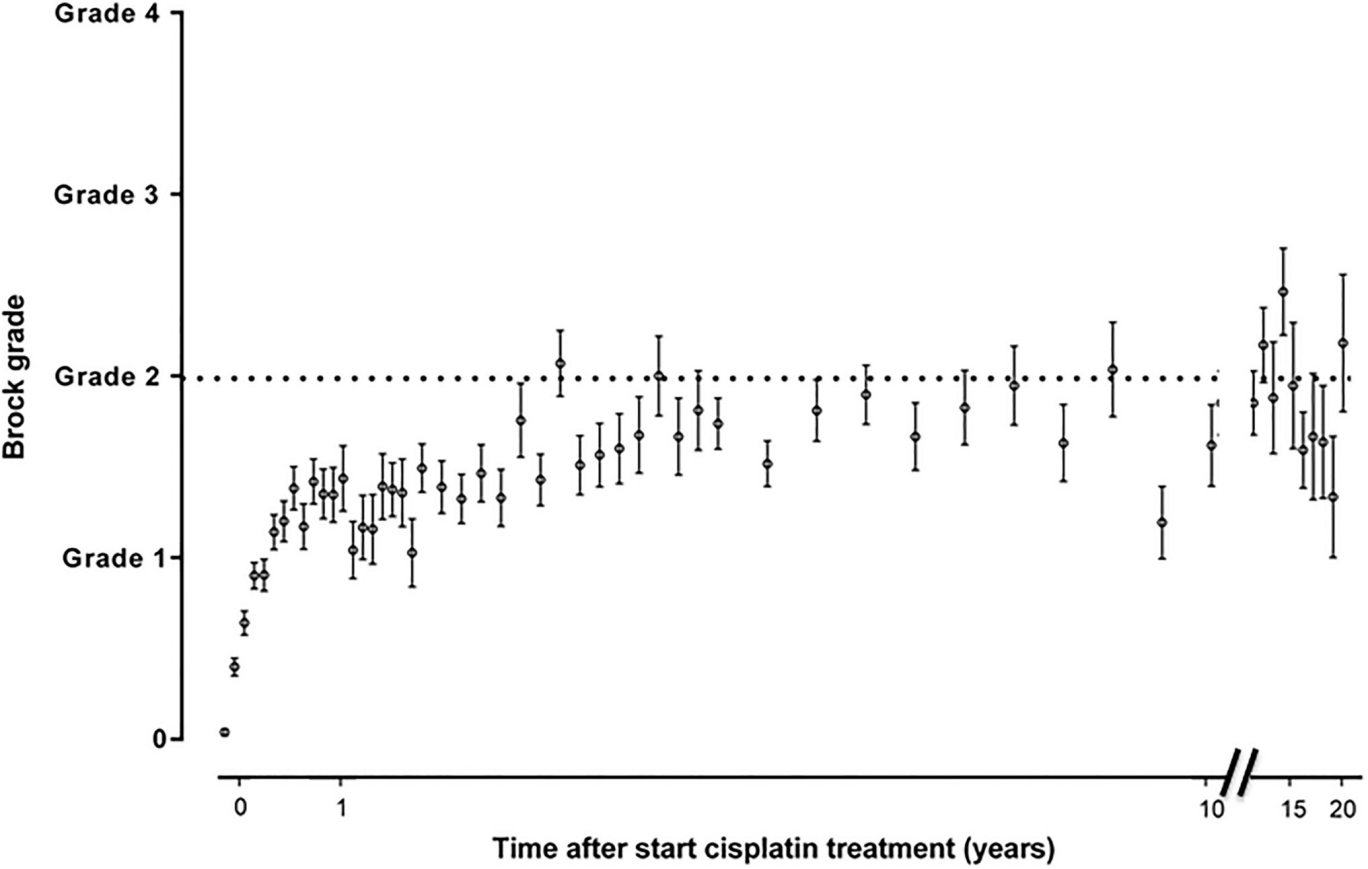
Progression of hearing loss based on Brock grade. The time after start cisplatin treatment in 654 patients is depicted on the x-axis and the hearing loss grade is depicted on the y-axis. The dots represent the mean and the whiskers represent the standard error of the mean ototoxicity grade. The dotted horizontal line depicts the cut-off for hearing loss yes/no.

**Fig 4:**
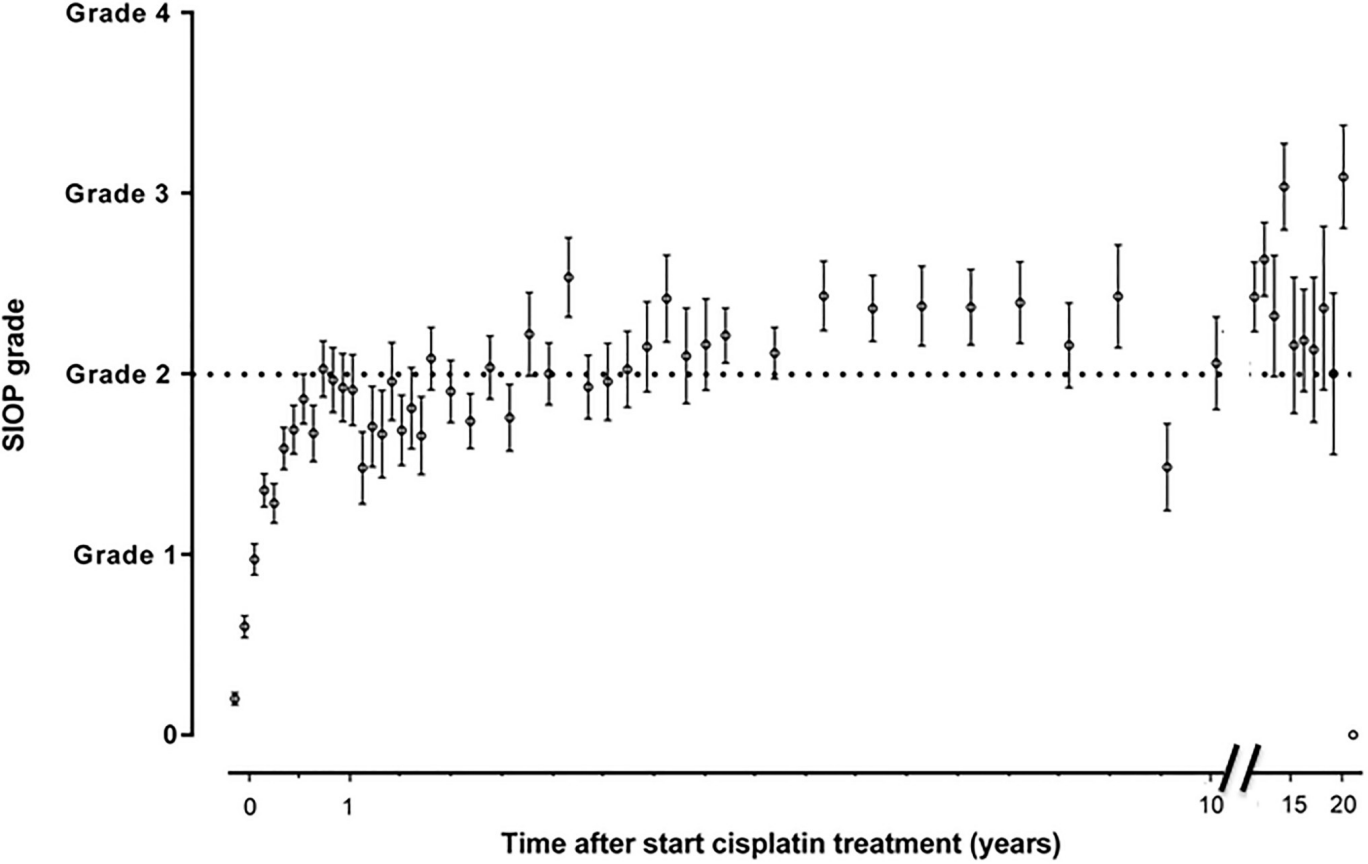
Progression of hearing loss based on SIOP grade. The time after start cisplatin treatment in 654 patients is depicted on the x-axis and the hearing loss grade is depicted on the y-axis. The dots represent the mean and the whiskers represent the standard error of the mean ototoxicity grade. The dotted horizontal line depicts the cut-off for hearing loss yes/no.

**Fig 5:**
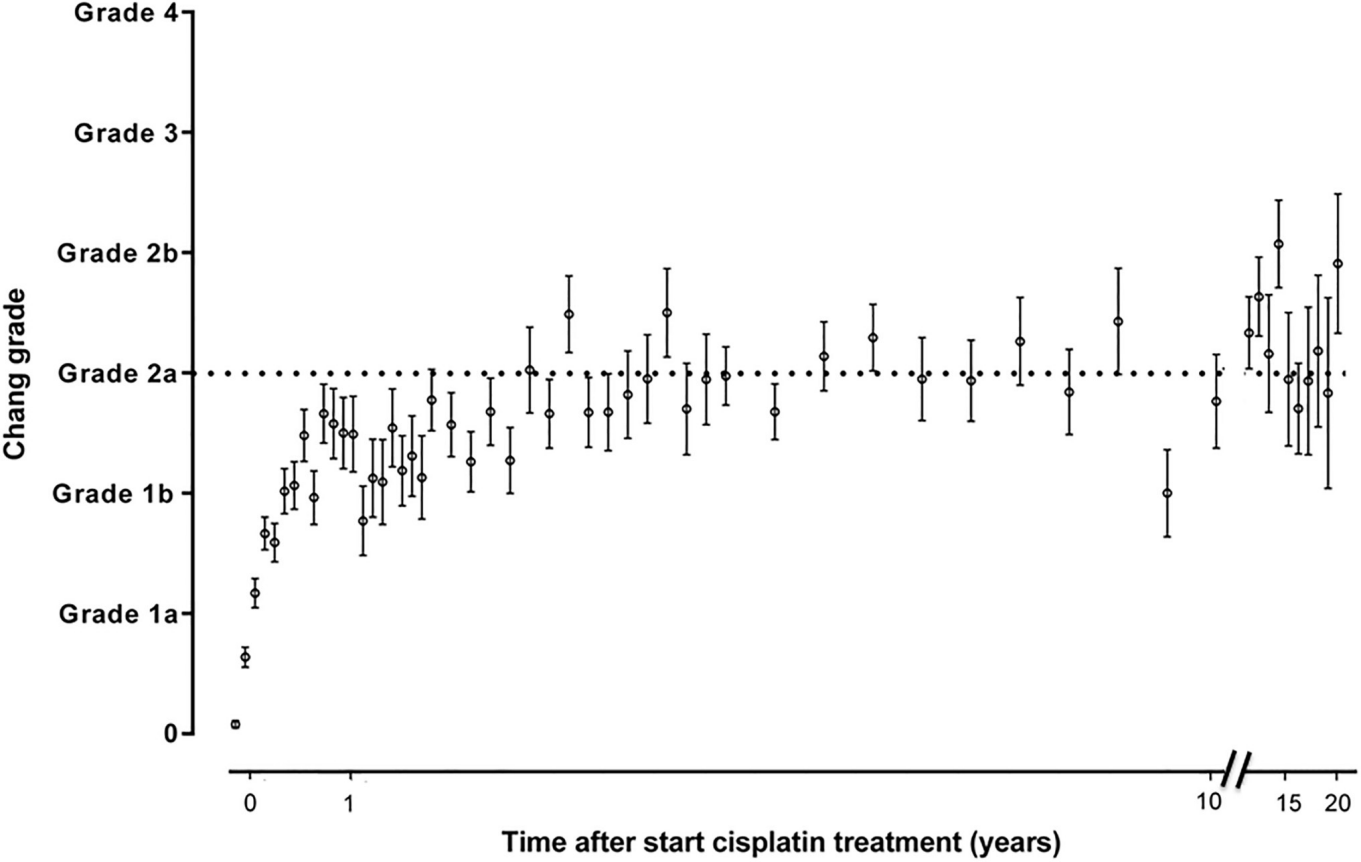
Progression of hearing loss based on Chang grade. The time after start cisplatin treatment in 654 patients is depicted on the x-axis and the hearing loss grade is depicted on the y-axis. The dots represent the mean and the whiskers represent the standard error of the mean ototoxicity grade. The dotted horizontal line depicts the cut-off for hearing loss yes/no.

Figs 2–5 show the sequential audiometric measurements. When evaluating the time to detection of deleterious hearing loss, on average, Muenster detected hearing loss the earliest in time, followed by Chang, Brock and SIOP. The overall trend of hearing function was similar between the different grading scales: hearing function decreased over time, stabilized at long-term follow-up but showed no recovery.

## Discussion

Several grading scales have been developed for the purpose of describing hearing loss in pediatric oncology patients. In this study, the application of five commonly used ototoxicity grading scales was compared in a large cohort of cisplatin treated childhood cancer patients, namely: Brock, Chang, Muenster, SIOP Boston and CTCAEv4.03. The scales were applied to 3,799 audiograms of 654 platinum-treated pediatric patients and a generally good concordance between the scales was observed.

The Brock scale is the oldest ototoxicity scale that was developed taking into account the course of high to low frequency loss[19]. Platinum agents first affect hearing sensitivity for the high frequency, that is high-pitched sounds and progressively affects the sound spectrum from high to lower frequencies, that is lower pitches[1]. In our study, a high degree of concordance was observed between Brock and Chang (ĸ = 0.975). The Chang scale[20] was developed as a modified version of the Brock scale which could explain the high concordance. Although Brock grade 2 and Chang grade 2a are similar in coding (hearing loss above or equal to 40 dB at 4 kHz and above), a major difference in the number of patients with grade 2 hearing loss is noted (Brock grade 2: 23.5% and Chang grade 2a: 9%). The reason for this discrepancy could be the incorporation the sub-scales in the Chang scale to capture hearing thresholds in the mild loss range of 20 to 40 dB at all frequencies. A similar number of patients having hearing loss were identified with Brock grade 4 (5.4%) and Chang grade 4 (5.5%, hearing loss above 40 dB at 2 kHz and above).

The SIOP Boston scale, validated on international multicenter audiological data[16], combined all elements of all previously published ototoxicity classification systems and was based on modification of the pediatric functional hearing loss scale of Lewis et al.[22]. Our study was in accordance with Bass et al.[23], showing a strong concordance between the Chang and SIOP scales. The observed concordance between SIOP and the other ototoxicity scales in our study was high as well.

Detection of early stage hearing loss and early prediction for the need of hearing support was the motivation for the Muenster scale[21]. This is in accordance with our study, showing that the Muenster scale detected deleterious hearing loss the earliest in time. Unfortunately, cochlear damage in the extended high frequencies and detection on formal testing is not reported by any of these grading scales. However, the American Speech-Language and Hearing Association (ASHA) definition of ototoxicity will capture ototoxicity in the extended high frequencies and is widely used among audiologists to help inform physicians that an adverse effect has occurred, well before the conventional frequency range is affected. Both baseline and repeat testing are required to monitor deterioration[24]. Although the Muenster scale was developed and validated using the Brock scale, concordance between the Muenster and Brock scale was low (ĸ = 0.641) in our study. Concordance between Muenster and Chang (ĸ = 0.636) was low as well, while Muenster and SIOP scales (ĸ = 0.744), and Muenster and CTCAEv4.03 (ĸ = 0.857) showed higher concordances.

The CTCAE requires baseline testing to assess changes of hearing function. Since actual baseline thresholds were often not available, we assumed baseline of 0 dB hearing loss at all frequencies, and used hearing threshold levels. Our study showed lower concordance between CTCAE version 4.03 and the Brock scale (ĸ = 0.681) than the study by Knight et al. (ĸ = 0.88)[8]. However, Knight et al. used version 3 of the CTCAE criteria and we have used adjusted CTCAE version 4.03 criteria based on absolute thresholds.

Within one year after end of treatment, 38% (Brock ≥grade 2), 38% (Chang ≥grade 2a), 62% (CTCAEv4.03 ≥grade 2), 44% (SIOP ≥grade 2) and 46% (Muenster ≥grade 2b) of patients had deleterious hearing loss. It is notable that ototoxicity may be occurring before the adverse drug effect is captured on a conventional audiogram which only tests up to 8000 Hz. Auditory access to the richness of the sound environment is diminished as a child losses hearing, first in the extended high frequency range, progressing into the conventional frequency range critical for understanding speech and for developing speech production and full language concepts[25]. Audiologists assess audiological needs for patients on an individual basis. Children will have significantly difficulty understanding language in the presence of background noise once their hearing threshold is greater than 20 dB at 6000 Hz and above. Typically, once the hearing threshold is greater than 20 dB at 3000 Hz and above in at least one ear, corresponding to SIOP grade 3, a hearing aid will be considered. Children who have hearing loss at 4000 or 6000 Hz and above may struggle with understanding language over distance or in the presence of background noise[5, 26]. In such cases hearing equipment involving remote microphone technology (e.g. a frequency modulation (FM) system) may be helpful.

In order to detect high frequency hearing loss at an early stage, extended high frequencies above 8 kHz should be measured. With high frequency audiometry, treatment protocols could be modified, or otoprotective medication such as sodium thiosulfate or amifostine could be administered[27, 28]. With early detection of ototoxic effects, aural rehabilitation and counseling can be introduced at an early stage during treatment. Of the evaluated scales, the Chang scale is the only scale to incorporate extended high frequency audiometry. Knight et al. demonstrated ototoxicity in 25% of cisplatin-treated pediatric patients who did not have ototoxicity in the conventional frequency range (up to 8000 Hz)[1]. Unfortunately the required equipment is not always available and is not part of standard practice[29]. In general, which classification system to use will depend on the purpose of the monitoring. If the purpose is to detect ototoxic change as early as possible during treatment, the Muenster classification is the most useful. Hence, on the basis of earliest time to detect hearing loss, and high concordance with other grading scales, as well as including progressive losses into the lower frequencies (0.25–1 kHz), we found a benefit for using the Muenster classification as the most optimal grading system for predicting clinically relevant ototoxicity during platinum treatment. This is supported by a study in a small subset of platinum-treated children by Lafay-Cousin et al. who described that, already after two courses of cisplatin in childhood cancer patients, Muenster criteria had a higher sensitivity (67%) and specificity (87%) to predict the need for a hearing aid after end of treatment compared to Brock, Chang, CTCAE and ASHA[30]. However, more studies are needed to find the optimal classification system that has a high correlation between the grading outcome and the functional outcome that is easy to apply and to understand.

A large sample size and audiological grading according to Brock, Chang, SIOP Boston and Muenster scales all performed by one researcher are strengths of this study. Since the ototoxicity grading systems limit grade definition to audiograms, although they may have reasonable concordance, they may be too simplistic for accurate phenotyping of hearing function. It can make a significant difference whether grading is based on audiogram alone or on audiological datasets that allow more effective elimination of other contributing factors to the hearing loss. By assessing a child’s overall hearing status, additional etiologies should be eliminated by taking into account as many factors as possible, as was the case for the CTCAEv4.03 criteria in our study.

The lack of standardized time points relative to platinum dosing for audiometric testing during and after treatment for pediatric cancer is not only a limitation that we have encountered in our study, but is a general limitation in pediatric oncology. Protocols are essential for prospective monitoring of hearing function in childhood cancer patients receiving treatment. Ideally, hearing status will be measured after each cycle of cisplatin, although the medical treatment plan may not be alterable, adjustment to the hearing loss as it unfolds can be helped with suitable audiological counselling.

In summary, we evaluated the concordance among five ototoxicity grading scales using Kappa values in a large cohort of pediatric patients that were treated with platinum chemotherapy. The analysis was not used to determine the best grading scale, but to compare the differences between them. Differences in concordances appeared to be related largely to differing distribution levels of severity of hearing loss, with some scales taking into account losses below 40 dB. Therefore, the classification scales are not interchangeable. Nevertheless, it is important to take into consideration that platinum-induced hearing loss, regardless which grading scale used, can have significant complications for children[6, 10]. In both psychosocial and developmental areas, difficulties can arise[5, 8, 31]. We observed that hearing function continues to decline with time and stabilizes at some point. This is in accordance with previous studies, showing that platinum concentrations are detectable in plasma up to 20 years after administration[32, 33]. At long-term follow-up in our study, no improvement of hearing function was observed.

It is our obligation as pediatric oncology professionals to consider the functional implications of hearing loss on educational and psychosocial scale[8, 34] and therefore, close monitoring of hearing function after starting cisplatin treatment needs to be conducted.

## Supporting information

S1 Fig U.S. National Cancer Institute Common Technology Criteria for Adverse Events version 4.03 (CTCAEv4.03) grading scale (A), Brock grading scale (B), Chang grading scale (C), Muenster grading scale (D) and SIOP grading scale (E). The yellow part depicts the area where hearing loss is defined.
(PDF)

S2 Fig Number of audiograms graded according to the U.S. National Cancer Institute Common Technology Criteria for Adverse Events version 4.03 (CTCAEv4.03), Brock, Chang, Muenster and SIOP grading scale. (PDF)
