# Supplementary material for: A comparison of the Muenster, SIOP Boston, Brock, Chang and CTCAEv4.03 ototoxicity grading scales applied to 3,799 audiograms of childhood cancer patients treated with platinum-based chemotherapy
Source: PLoS One. 2019 Feb 14;14(2):e0210646. doi: 10.1371/journal.pone.0210646 (PMC6375552; doi:10.1371/journal.pone.0210646)
Supplement: S1 Fig — The yellow part depicts the area where hearing loss is defined. (PDF) [file pone.0210646.s001.pdf]

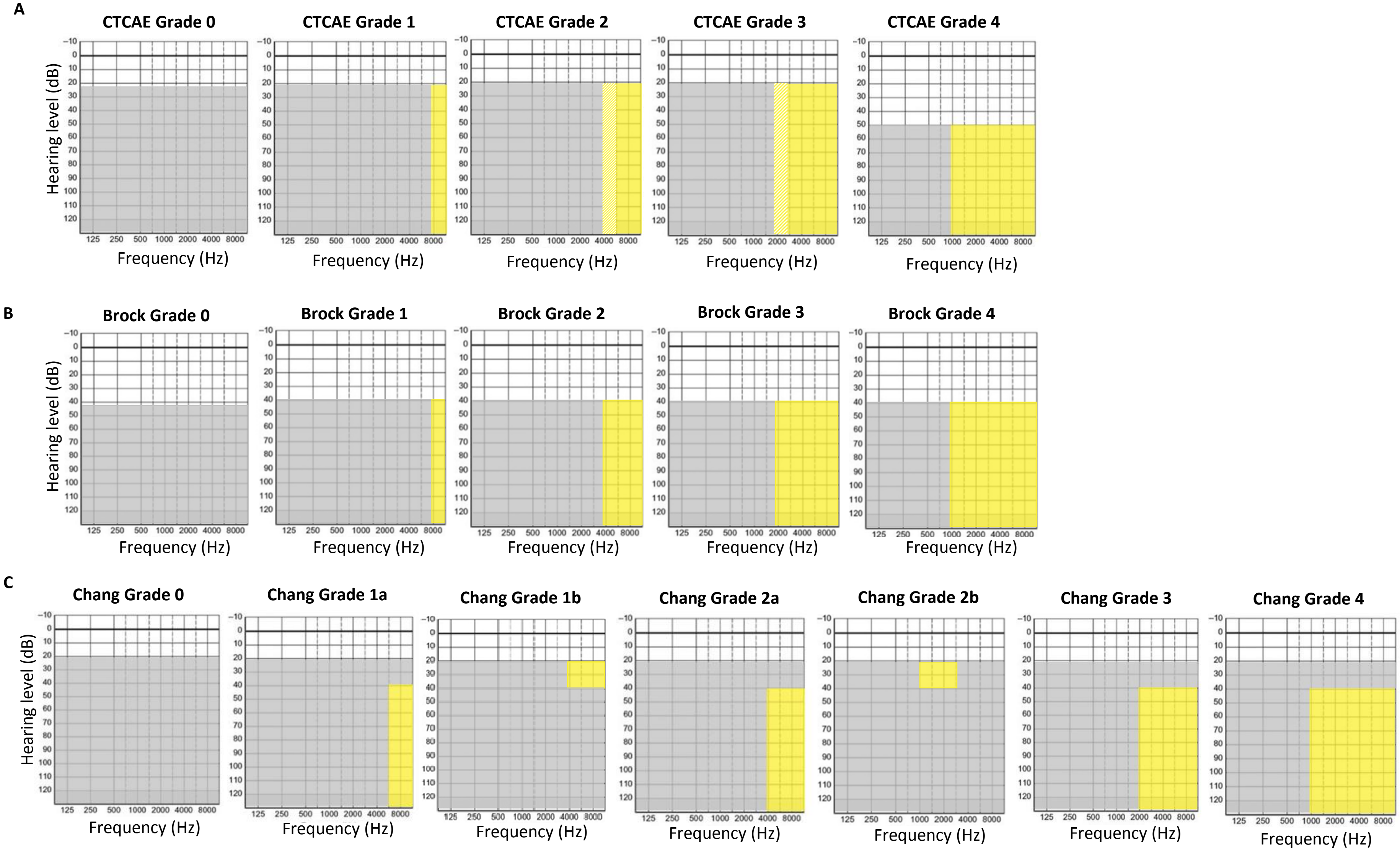
A
CTCAE Grade 0
CTCAE Grade 1
CTCAE Grade 2
CTCAE Grade 3
CTCAE Grade 4
B
Brock Grade 0
Brock Grade 1
Brock Grade 2
Brock Grade 3
Brock Grade 4
C
Chang Grade 0
Chang Grade 1a
Chang Grade 1b
Chang Grade 2a
Chang Grade 2b
Chang Grade 3
Chang Grade 4
Hearing level (dB)
−10 0 10 20 30 40 50 60 70 80 90 100 110 120
Frequency (Hz)
125 250 500 1000 2000 4000 8000

D

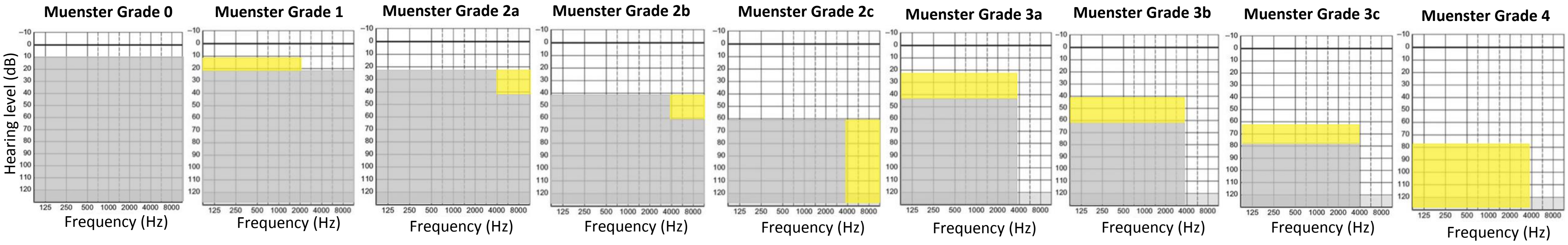

E

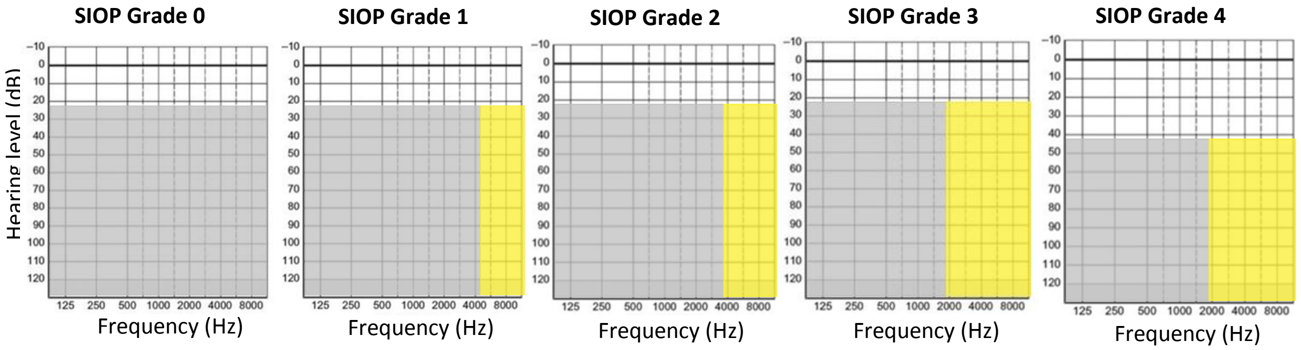

S1 Figure. Schematic overview of classification systems. U.S. National Cancer Institute Common Technology Criteria for Adverse Events version 4.03 (CTCAEv4.03) grading scale (**A**), Brock grading scale (**B**), Chang grading scale (**C**), Muenster grading scale (**D**) and SIOP grading scale (**E**). The yellow part depicts the area where hearing loss is defined.
