# Supplementary material for: A comparison of the Muenster, SIOP Boston, Brock, Chang and CTCAEv4.03 ototoxicity grading scales applied to 3,799 audiograms of childhood cancer patients treated with platinum-based chemotherapy
Source: PLoS One. 2019 Feb 14;14(2):e0210646. doi: 10.1371/journal.pone.0210646 (PMC6375552; doi:10.1371/journal.pone.0210646)
Supplement: S2 Fig — (PDF) [file pone.0210646.s002.pdf]

	SIOP Boston						
		**0**	**1**	**2**	**3**	**4**	**Total**
	**0**	744	9	1	0	0	**754**
	**1**	510	20	9	1	0	**540**
	**2a**	36	310	58	22	2	**428**
	**2b**	5	261	139	45	9	**459**
**Muenster**	**2c**	0	139	309	42	8	**498**
	**3a**	0	1	11	263	21	**296**
	**3b**	0	0	2	246	185	**433**
	**3c**	0	0	0	63	145	**208**
	**4**	0	0	0	4	174	**187**
	**Total**	**1295**	**740**	**529**	**687**	**543**	**3794**

	Brock						
		**0**	**1**	**2**	**3**	**4**	**Total**
	**0**	754	0	0	0	0	**754**
	**1**	536	2	1	1	0	**540**
	**2a**	319	88	13	3	2	**425**
	**2b**	3	359	88	6	6	**462**
**Muenster**	**2c**	1	232	258	1	5	**497**
	**3a**	1	27	220	32	12	**292**
	**3b**	0	1	234	160	38	**433**
	**3c**	0	1	55	126	26	**208**
	**4**	0	0	1	83	94	**178**
	**Total**	**1614**	**710**	**870**	**412**	**183**	**3789**

	Chang								
		**0**	**1a**	**1b**	**2a**	**2b**	**3**	**4**	**Total**
	**0**	753	0	1	0	0	0	0	**754**
	**1**	53	3	1	1	0	1	0	**539**
	**2a**	290	80	29	4	18	2	3	**426**
	**2b**	5	265	91	51	31	13	6	**462**
**Muenster**	**2c**	0	147	85	222	26	11	6	**497**
	**3a**	0	2	21	3	154	98	14	**292**
	**3b**	0	1	1	0	1	389	41	**433**
	**3c**	0	0	0	0	1	175	32	**208**
	**4**	0	0	0	0	0	81	97	**178**
	**Total**	**1581**	**498**	**229**	**281**	**231**	**770**	**199**	**3789**

	Brock						
		**0**	**1**	**2**	**3**	**4**	**Total**
	**0**	1287	6	1	1	0	**1295**
	**1**	271	466	2	1	0	**740**
**SIOP**	**2**	44	185	291	4	1	**525**
	**3**	12	51	555	57	7	**683**
	**4**	0	1	19	349	174	**543**
	**Total**	**1614**	**709**	**869**	**412**	**182**	**3786**

	Chang								
		**0**	**1a**	**1b**	**2a**	**2b**	**3**	**4**	**Total**
	**0**	1283	9	1	1	1	0	0	**1295**
	**1**	265	465	8	0	1	1	0	**740**
**SIOP**	**2**	27	20	188	268	16	6	1	**526**
	**3**	6	3	30	12	211	408	12	**682**
	**4**	0	1	0	0	2	355	185	**543**
	**Total**	**1581**	**498**	**227**	**281**	**231**	**770**	**198**	**3786**

	Chang								
		**0**	**1a**	**1b**	**2a**	**2b**	**3**	**4**	**Total**
**Brock**	**0**	1560	22	22	3	6	0	0	**1613**
	**1**	17	471	195	1	24	2	0	**710**
	**2**	3	3	10	277	198	372	6	**869**
	**3**	1	2	1	0	2	394	12	**412**
	**4**	0	0	0	0	0	2	181	**183**
	**Total**	**1581**	**498**	**228**	**281**	**230**	**770**	**199**	**3787**

	Muenster										
		**0**	**1**	**2a**	**2b**	**2c**	**3a**	**3b**	**3c**	**4**	**Total**
**CTCAEv4.03**	**0**	105	62	3	0	1	0	0	0	0	**171**
	**1**	0	0	23	19	7	0	0	0	1	**50**
	**2**	0	0	7	55	60	7	1	1	2	**133**
	**3**	0	0	0	0	1	43	59	27	24	**154**
	**4**	0	0	0	0	0	0	1	1	12	**14**
	**Total**	**105**	**62**	**33**	**74**	**69**	**50**	**61**	**29**	**39**	**522**

	SIOP Boston						
		**0**	**1**	**2**	**3**	**4**	**Total**
**CTCAEv4.03**	**0**	166	4	0	0	1	**171**
	**1**	0	48	1	0	1	**50**
	**2**	1	48	71	6	6	**132**
	**3**	0	0	0	91	63	**154**
	**4**	0	0	0	0	14	**14**
	**Total**	**167**	**100**	**72**	**97**	**85**	**521**

	Brock						
		**0**	**1**	**2**	**3**	**4**	**Total**
**CTCAEv4.03**	**0**	170	0	1	0	0	**171**
	**1**	17	31	1	0	1	**50**
	**2**	4	73	51	2	3	**133**
	**3**	0	4	78	57	14	**153**
	**4**	0	0	0	3	11	**14**
	**Total**	**191**	**108**	**131**	**62**	**29**	**521**

	Chang								
		**0**	**1a**	**1b**	**2a**	**2b**	**3**	**4**	**Total**
**CTCAEv4.03**	**0**	170	0	0	0	0	1	0	**171**
	**1**	16	32	0	1	0	0	1	**50**
	**2**	3	48	27	43	5	4	3	**133**
	**3**	0	0	3	0	27	108	15	**153**
	**4**	0	0	0	0	0	3	11	**14**
	**Total**	**189**	**80**	**30**	**44**	**32**	**116**	**30**	**521**

S2 Figure. Cross tables of classification systems. Number of audiograms graded according to the U.S. National Cancer Institute Common Technology Criteria for Adverse Events version 4.03 (CTCAEv4.03), Brock, Chang, Muenster and SIOP grading scale.
